# Case Report: A rare homozygous patient affected by *TTR* systemic amyloidosis with a prominent heart involvement

**DOI:** 10.3389/fcvm.2023.1164916

**Published:** 2023-08-29

**Authors:** Emanuele Micaglio, Gloria Santangelo, Silvia Moscardelli, Daniela Rusconi, Francesco Musca, Alessandro Verde, Laura Campiglio, Francesca Bursi, Marco Guazzi

**Affiliations:** ^1^ Arrhythmia and Electrophysiology Department, IRCCS Policlinico San Donato, Milan, Italy; ^2^ Division of Cardiology, Department of Health Sciences, San Paolo Hospital, University of Milan, Milan, Italy; ^3^ Pathological Anatomy, Cytogenetics, Molecular Pathology, San Paolo Hospital, ASST Santi Paolo and Carlo, Milan, Italy; ^4^ UO Cardiologia 4, ASST Grande Ospedale Metropolitano Niguarda, Milan, Italy; ^5^ Clinical Neurology Unit, ASST Santi Paolo e Carlo, Department of Neuroscience, University of Milan, Milan, Italy

**Keywords:** hereditary transthyretin amyloidosis cardiomyopathy, homozygous variant, Val142Ile, atrial fibrillation, stroke

## Abstract

Hereditary transthyretin amyloidosis is a severe, adult-onset autosomal dominant inherited systemic disease predominantly affecting the peripheral and autonomic nervous system, heart, kidney, and the eyes. We present a case of a Caucasian 65-year-old man with cardiac amyloidosis and the homozygous mutation Val142Ile (classically, Val122Ile) in the transthyretin gene. We provide a genotype-phenotype correlation regarding the genetic status of both heterozygous and homozygous individuals and their clinical conditions at the time of genetic testing.

## Case

A 65-year-old man presented for the first time to our hospital when he was admitted to the stroke unit because of subacute ischemic stroke in left middle cerebral artery territory. Before admission, the patient showed aphasia, right hemianopia, and right-sided hemiparesis affecting the arm, which spontaneously improved by hospital arrival.

## Medical history

His medical history was remarkable for chronic ischemic heart disease and previous off-pump coronary bypass. The perioperative course was complicated with paroxysmal atrial fibrillation (AF) treated with pharmacologic cardioversion. At that time, anticoagulant therapy was not deemed necessary due to a CHA_2_DS_2_VASc = 1. An infrarenal aortic aneurysm was incidentally discovered and surgically treated with placement of aorto-bi-iliac prosthesis at age 62. The patient was followed up at another center until index hospitalization.

## Differential diagnosis

The patient was investigated to search for embolic sources in the suspect of recurrent paroxysmal AF, ischemic heart disease or cardiomyopathy, including metabolic disorders, and either wild-type or mutant transthyretin cardiac amyloidosis.

## Investigation

The first computed tomography (CT) showed ischemic stroke in the territory of the left middle cerebral artery with hemorrhagic transformation contraindicating the initiation of anticoagulant therapy and necessitating initial suspension of antiplatelet therapy.

The electrocardiogram showed sinus rhythm, 75 beats/minute heart rate, normal PR, normal QRS voltages, pseudo-*Q*-waves in inferior leads and aspecific *T*-wave abnormalities. The echocardiographic examination revealed severe increase of the left ventricular (LV) mass indexed to the body surface area (148 g/m^2^) and marked increase of septal and lateral ventricular wall thicknesses (16 and 15 mm, respectively) with a myocardial texture highly retractile and inhomogeneous. LV ejection fraction was moderately reduced (43%) as well as global longitudinal strain (GLS) (−14%) with a base to apex gradient. The examination showed also severe left atrial (LA) dilation, thickening of the interatrial septum (9 mm) and grade II diastolic dysfunction, low mitral annulus e′ velocities on tissue Doppler imaging, and high E/e′. Right ventricle showed normal size, thickness, and systolic function. There was a mild tricuspid regurgitation, and pulmonary artery systolic pressure was 43 mmHg. As a collateral, three small left-to-right shunts due cribrosus foramen ovale were observed. These examinations raised the suspicion of an underlying deposit disease and further examinations were performed. Blood count and biochemical findings were normal, with no proteinuria, NT-pro-BNP was 1,966 pg/ml, high sensitivity-Troponin I was 0.064 ng/ml (normal reference below 0.036 ng/ml), normal cupremia was 91 μg/dl, and creatinine kinase was 39 U/L. The serum immunoelectrophoretic pattern was normal, no free chains of immunoglobulin were detected in the blood or urine, and this excluded the diagnosis of amyloidosis light-chain amyloidosis.

**Figure 1:**
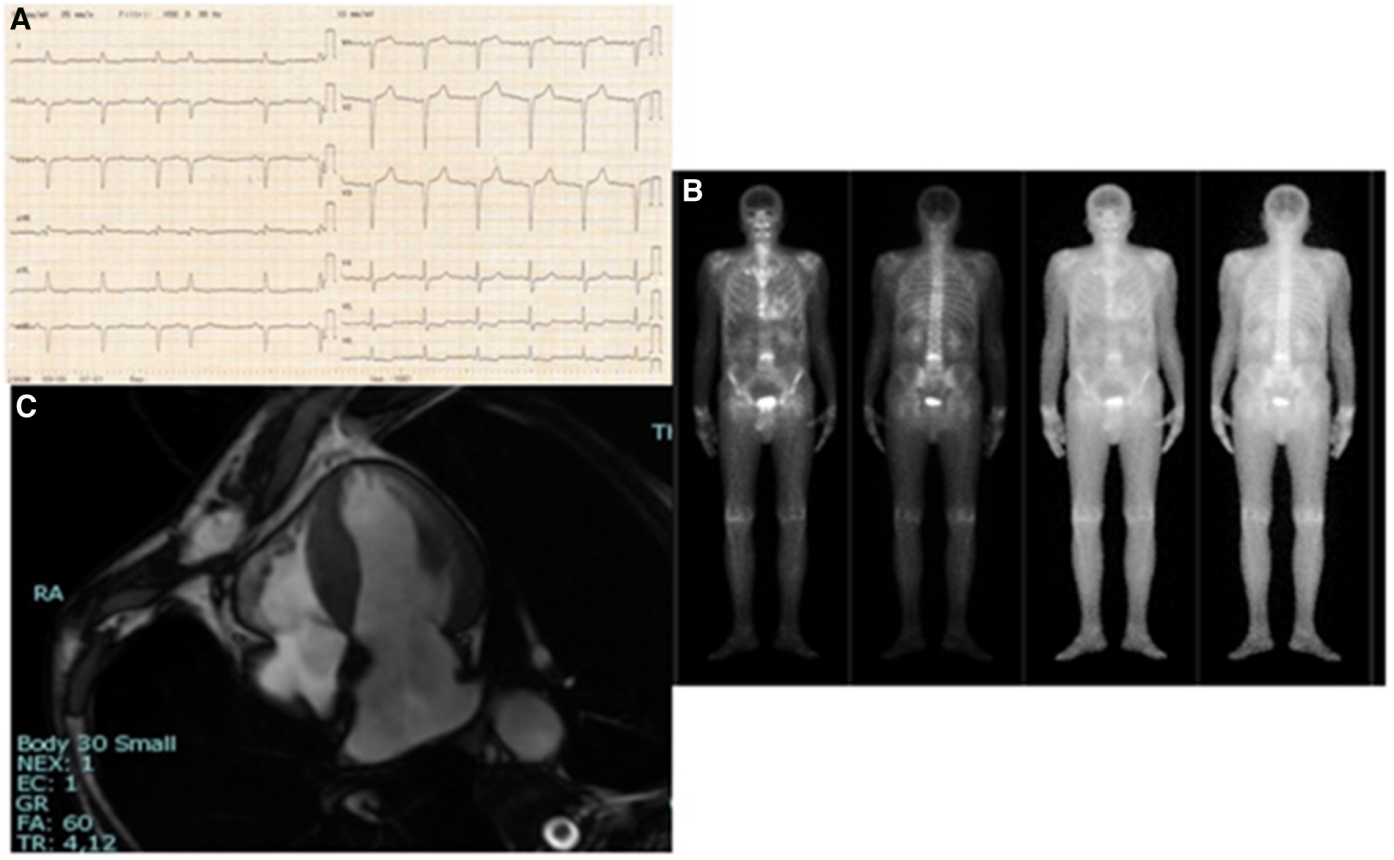
(**A**) 12-lead electrocardiogram: sinus rhythm, heart rate 75/min, normal PR, normal QRS voltages, pseudo-*Q-*waves in inferior leads and aspecific *T-*wave abnormalities. (**B**) Four-chamber cine cardiovascular magnetic resonance: increased wall thickness; right ventricle normal in size and function. (**C**) Total body scintigraphy with Technetium-99m oxidronate: radiotracer deposit in the LV greater than bone intensity.

**Figure 2:**
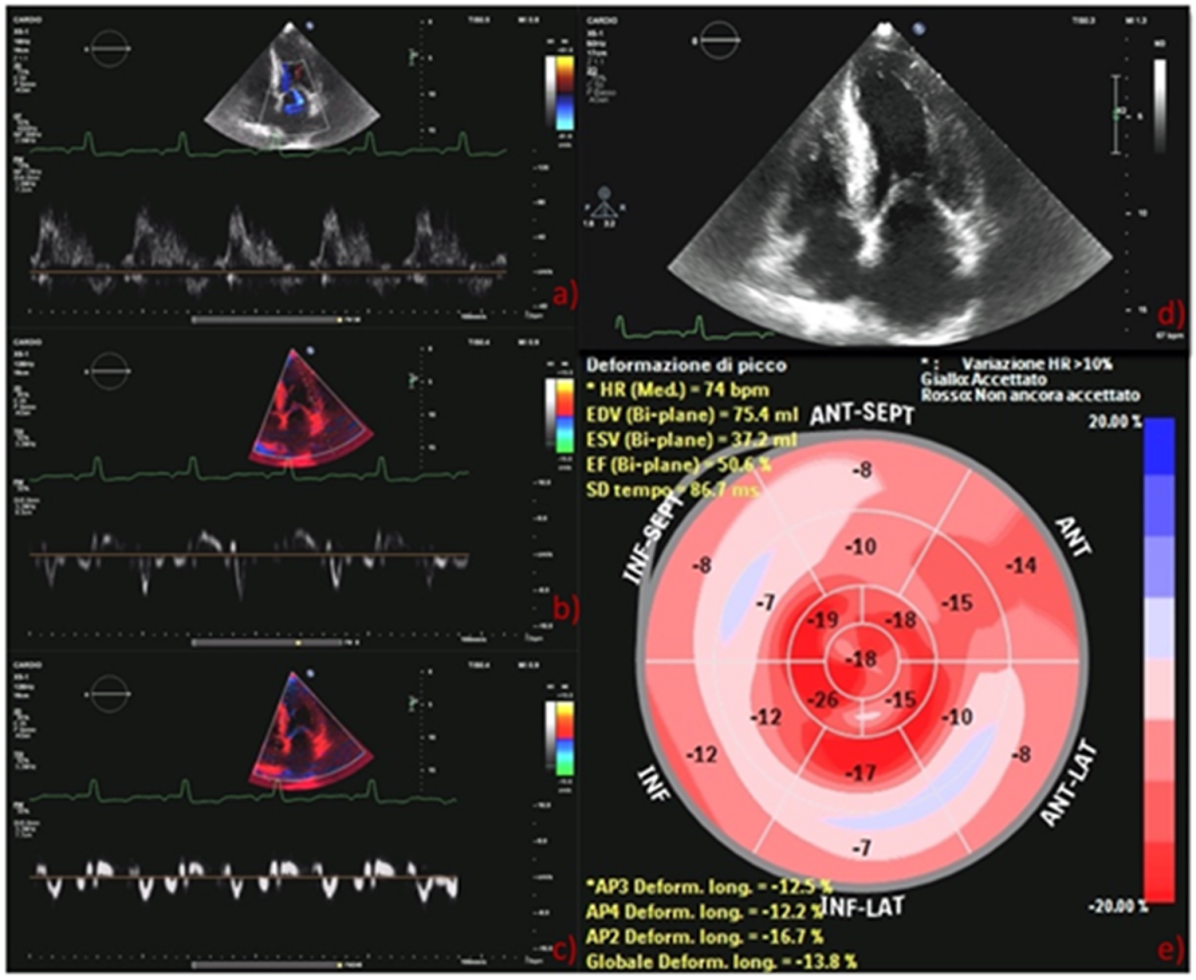
(**A**) Pulsed mitral wave Doppler: grade II diastolic dysfunction with elevation E/A ratio (1.93); (**B**) lateral tissue Doppler imaging: low mitral annulus e′ velocities (8 cm/s); (**C**) septal tissue Doppler measurement: low mitral annulus e′ velocities (6 cm/s); (**D**) GLS bull's eye diagram: typical apical sparing of amyloidosis. GLS, global longitudinal strain.

On total body scintigraphy with Technetium-99m oxidronate, radiotracer deposit in the LV was greater than bone intensity, confirming the suspicion of transthyretin (TTR) amyloid cardiomyopathy. Cardiac magnetic resonance imaging accordingly showed diffuse increased wall thickness, no LV enlargement, and mildly reduced ejection fraction. In Figures 1, 2, we summarize the patient's findings at imaging investigations.

To characterize the type of TTR amyloidosis, a genetic molecular test was performed.

Genomic DNA was isolated from whole peripheral blood of the proband by using an automatic DNA Extractor QIAcube (Qiagen) following the manufacturer's protocol, and subsequently being quantified using Qubit dsDNA BR assay kit (Life Technologies). Exons 1, 2, 3, and 4 and 40 nucleotides covering the intronic regions at the beginning and at the end of each exon of the *TTR* gene (NM_000371.3) were amplified by polymerase chain reaction. Subsequently, DNA fragments were directly sequenced using an ABI Prism 3130 automated sequencer.

**Figure 3:**
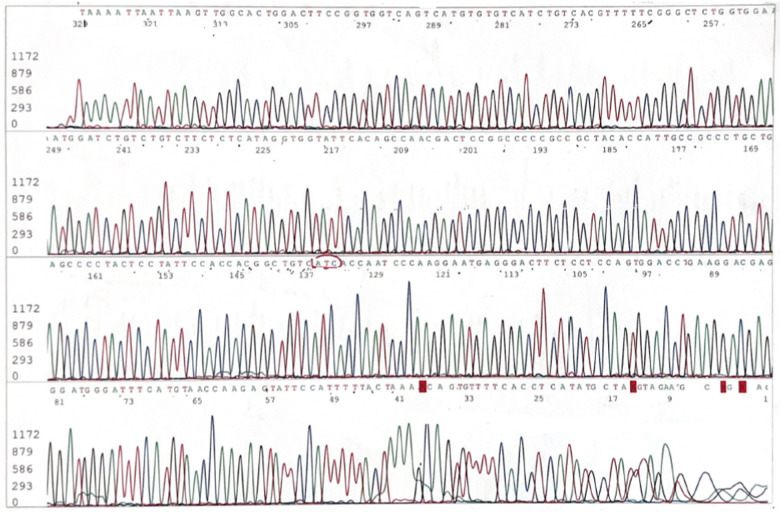
Electropherogram. Homozygous mutation in the *TTR* gene.

Molecular examination revealed the presence of a homozygous variant in the exon 4 of the *TTR* (NM_000371.4) gene: c.424G > A, p.Val142Ile (formerly Val122Ile), *rs76992529*. In Figure 3, we show the presence of homozygous mutation in the *TTR* gene at electropherogram.

This variant is very well known as pathogenic because it has been already published several times in patients clinically affected by TTR amyloidosis. Moreover, the variant was further checked using VarSome (11.3 version) classification and searched in the Mutation in Hereditary Amyloidosis Database that reported all the pathogenic *TTR* mutations including this one ((https://databases.lovd.nl/shared/genes/TTR). We were not able to test for ancestry informative markers to absolutely verify the lack of African ancestry. Of note, due to the patients’ phenotype consistent with Caucasian ethnicity, we considered redundant to exclude any possible African ancestry using the haplotype approach as well. We were not able to examine the proband's mother because she lives in another region.

## Management

At post-discharge follow-up, repeated control brain CTs continued to show signs of hemorrhage for several months. Only 6 months later, after a negative control CT and neurological revaluation, edoxaban 60 mg was started considering stroke occurrence during antiplatelet therapy, current CHA_2_DS_2_VASc = 4, and history of AF.

The 6-minute walk-test distance was 360 m, indicating a slightly reduced functional capacity compared with general population (values between 400 and 700 m in 6 min).

The patient did not show any neurological symptoms, but detailed neurophysiological evaluation showed bilateral moderate nerve entrapment at the wrist (right > left) and length-dependent axonal sensory neuropathy (familiar amyloidosis polyneuropathy I).

The proband was sent to the regional referral center for specific therapy administration and was considered eligible for Patisiran administration. The clinical indication for this treatment was the presence of amyloidosic neuropathy in association with heart involvement (1). Patisiran was started at the dose of 300 μg per kg with intravenous infusion every 3 weeks and supplementation of 2,500 IU vitamin A per day.

At 2 months from the start of TTR silencing therapy, the patient is in stable clinical condition and therapy has been well tolerated. Other therapies include beta-blockers, statin, angiotensin-converting enzyme inhibitors, diuretic, and proton pump inhibitors. The reason for both beta-blockers/ACE inhibitor use was the history of abdominal aortic aneurysm and grade 1 arterial hypertension (150/100 mmHg) in several medical measurements. During this therapy, patient's heart rate has been 85 beats per minute without any detectable atrioventricular conduction delay.

**Figure 4:**
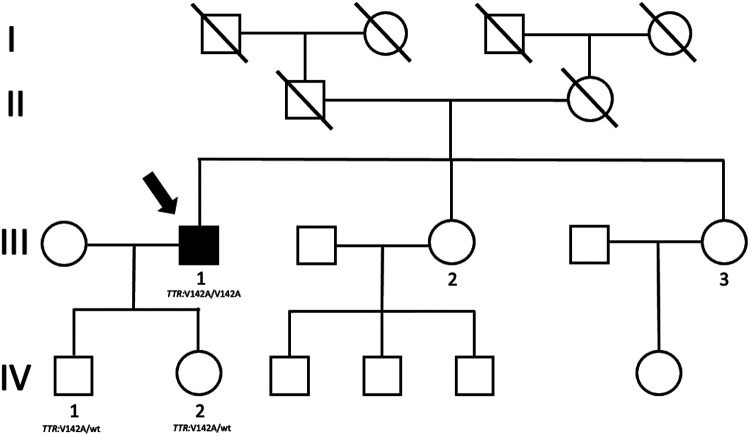
Proband's family tree. The arrow shows the proband who harbors mutation and clinical phenotype.

Family history was unremarkable; especially it was silent for cardiac diseases. No consanguinity was declared. The father of the patient died years ago for extracardiac causes, while his 86-year-old mother is still alive and has no symptoms or significant disease. The patient has two sisters aged 60 and 50 years living in another region. Both declared to be free of symptoms. Genetic testing and imaging screening was offered, but they preferred to pursue clinical and genetic investigations in the region where they live. The genetic analysis was extended to the 27-year-old son and 20-year-old daughter of the proband and, as expected, both harbor in heterozygous status the same mutation identified in the father (Figure 4). Clinical examination, electrocardiogram, echocardiogram, and electromyography showed normal findings in both of them.

## Discussion

Hereditary transthyretin amyloidosis variant (ATTRv) caused deposition of variant TTR protein.

ATTRv is characterized by an autosomal dominant pattern of inheritance and a variable range of phenotypes, form polyneuropathy, to cardiomyopathy, to renal and ocular involvement (2).

To date, 140 different *TTR* mutations have been identified, with the Val30Met (p.Val50Met) variant being the most frequent (2). In particular, this variant has been reported in 451 out of 26,062 subjects of African-American ancestry; the p.Val142Ile mutation observed in our proband is typically found in patients with African ancestry (3).

Therefore, according to the current literature, the Val142Ile carriers represent 2%–4% of the black population and 0.3%–1.6% in the general population, where it predominantly presents as a cardiomyopathy phenotype with little neurological involvement and with a late-onset clinical penetrance (4).

It is remarkable that our proband is Caucasian without declared African origin and his parents are not consanguineous (5). The patient is originally from Sicily, an Italian Southern region. We do not know if historical migration in this area might have influenced the distribution of these alleles, several dominations characterized Sicily history, including Greek, Normans, Turks, and Africans.

The Val142Ile mutation is extremely rare in Caucasian patients with few cases reported of ATTRv amyloidosis. However, Cappelli et al. described 5 unrelated patients out of 33 subjects with ATTRv (15.3%) with this mutation and phenotypic manifestations similar to those of African ancestry, hypothesizing that theVal142Ile pathogenic variant is not only an African ancestry mutation but could be also an underestimated Caucasian variant (6).

To date, among Caucasians, a small number of cases have been published with Val142Ile in homozygous state (https://www.ncbi.nlm.nih.gov/clinvar/variation/VCV000013426.47).

Reddi et al. found that the mean age of diagnosis in homozygotes was 64 ± 6 years, almost 10 years younger than heterozygotes; this corresponds to the age of our patient (7).

Of note, *TTR* mutations can display age-dependent penetrance that is valid in our case as well since heterozygous carriers of Val142Ile in their twenties did not show an overt phenotype yet.

Functional studies regarding this substitution demonstrated that the mutation causes an altered speed of tetramer dissociation, greater formation of amyloid fibrils, and an unstable TTR tetramer compared to wild type (8).

Based on the collective evidence, the Val142Ile variant is classified as pathogenic for familial transthyretin amyloidosis.

A systematic review showed that patients with Val142Ile cardiomyopathy are typically men, in the seventh-to eighth-decade of life, in 25%–38% diagnosed with AF. This variant was described associated with a longitudinally worse quality of life and a lower adjusted survival compared to wild-type TTR (4). Of note, the initial clinical presentation of our patient was stroke, and we do not know if it is due to AF or paradoxical embolism or other reason. AF is a frequent complication of amyloidosis, and the presence of amyloid cardiomyopathy in a patient with AF is an indication for anticoagulation regardless of CHA_2_DS_2_VASc score (9).

A prompt diagnosis of ATTRv cardiomyopathy is crucial for the initiation of effective drug treatments that can slow the progression of the disease or even improve cardiac function, as well as quality of life and functional outcomes. Of note, the positive outcome of patisiran therapy has been demonstrated by the so-called Apollo B study in terms of both NT-pro BNP reduction and GLS improvement in a median follow-up of 18.7 months (10) Some emerging therapies that are now available for ATTRv patients are mostly effective if initiated in early stages.

## Conclusions

The combination of biochemical and cardiac examinations supported by molecular characterization allowed the early diagnosis of the disease in our case. Moreover, by extending analysis to family members, it is possible to discover asymptomatic patients who, if harboring a pathogenic *TTR* variant, could be monitored during their lifetime preventing an occasional finding of the disease. High level of clinical suspicion is essential for early diagnosis, and a multidisciplinary coordinated work is critical to characterize the disease and stratify risk in order to rapidly initiate therapies that are now available and can effectively improve the disease course perhaps avoiding premature fatal event.

## Aim of this work

• To recognize that early clinical suspect and multidisciplinary work is essential for an early diagnosis of cardiac amyloidosis.
• To highlight that amyloid cardiomyopathy due to Val142Ile mutant TTR may be seen in Caucasian and to describe a rare case of homozygous.
• To understand that early diagnosis and categorization of TTR mutation allows initiation of specific therapies that are now available that can improve the course of the disease.

## Data Availability

The datasets presented in this study can be found in online repositories. The names of the repository/repositories and accession number(s) can be found in the article.
